# Antithrombotic Therapy in Coronary Artery Aneurysm, Ectasia, and Slow Flow: A Systematic Review

**DOI:** 10.1002/clc.70471

**Published:** 2026-09-25

**Authors:** Nasim Kheirandish, Maliheh Kamali, Alireza Khodadadiyan, Helia Bazroodi, Mohammadhossein Imani, Sahar Ghahramani, Amirhossein Saem, Amirhossein Imani, Haniyeh Alaei, Alireza Abdi‐Ardekani, Saideh Jamshidi, Mehdi Bazrafshan, Alireza Arzhangzade, Zahra Hooshanginezhad, Hamed Bazrafshan Drissi

**Affiliations:** ^1^ Cardiovascular Research Center Shiraz University of Medical Sciences Shiraz Iran; ^2^ Fellow of Echocardiography Cardiologist Shiraz University of Medical Sciences Shiraz Iran; ^3^ Student Research Committee, School of Medicine Shiraz University of Medical Sciences Shiraz Iran; ^4^ Department of Radiology Shiraz University of Medical Sciences Shiraz Iran; ^5^ Interventional Cardiologist Shiraz University of Medical Sciences Shiraz Iran; ^6^ Department of Cardiology, School of Medicine Shiraz University of Medical Sciences Shiraz Iran

**Keywords:** antithrombotic therapy, coronary artery aneurysm, coronary artery ectasia, coronary slow flow, dual antiplatelet therapy, oral anticoagulant

## Abstract

**Background:**

Coronary artery aneurysm (CAA), coronary artery ectasia (CAE), and coronary slow flow phenomenon (CSFP) are coronary pathologies associated with altered hemodynamics and increased thrombotic risk. However, optimal antithrombotic management remains uncertain because of limited and heterogeneous evidence.

**Objectives:**

This systematic review aimed to synthesize data on the efficacy and safety of antiplatelet and anticoagulant therapies in patients with CAA, CAE, and CSFP.

**Methods:**

Following PRISMA 2020 guidelines, PubMed, Scopus, Web of Science, and ProQuest were searched through September 2025 for randomized and observational studies evaluating antithrombotic therapy in adults with these conditions. Outcomes included major adverse cardiovascular events and major bleeding. Narrative synthesis was conducted using the SWiM framework. Risk of bias was assessed using ROBINS‐I and RoB 2.0, and certainty of evidence using GRADE.

**Results:**

Eleven studies comprising 3271 participants were included: six on CAE (*n* = 895), four on CAA (*n* = 2356), and one on CSFP (*n* = 20). In CAE, dual antiplatelet therapy (DAPT) and oral anticoagulation (OAC) were associated with fewer ischemic events than no therapy, although direct comparisons were inconsistent. The only randomized trial in CAE showed numerically lower recurrent myocardial infarction and cardiovascular death with single antiplatelet therapy (SAPT) plus direct oral anticoagulant (DOAC) versus DAPT, but findings were nonsignificant and based on few events. Evidence for CAA and CSFP remains limited and inconclusive.

**Conclusions:**

Intensified antithrombotic therapy may benefit patients with CAE, but evidence remains inconclusive for CAA and CSFP. Large, multicenter randomized trials are warranted to establish condition‐specific treatment recommendations.

## Graphical abstract

Antithrombotic therapy for coronary artery aneurysm, coronary artery ectasia, and coronary slow‐flow phenomenon remains insufficiently defined. This review highlights limited and heterogeneous evidence, supporting individualized treatment decisions and the need for stronger comparative studies.

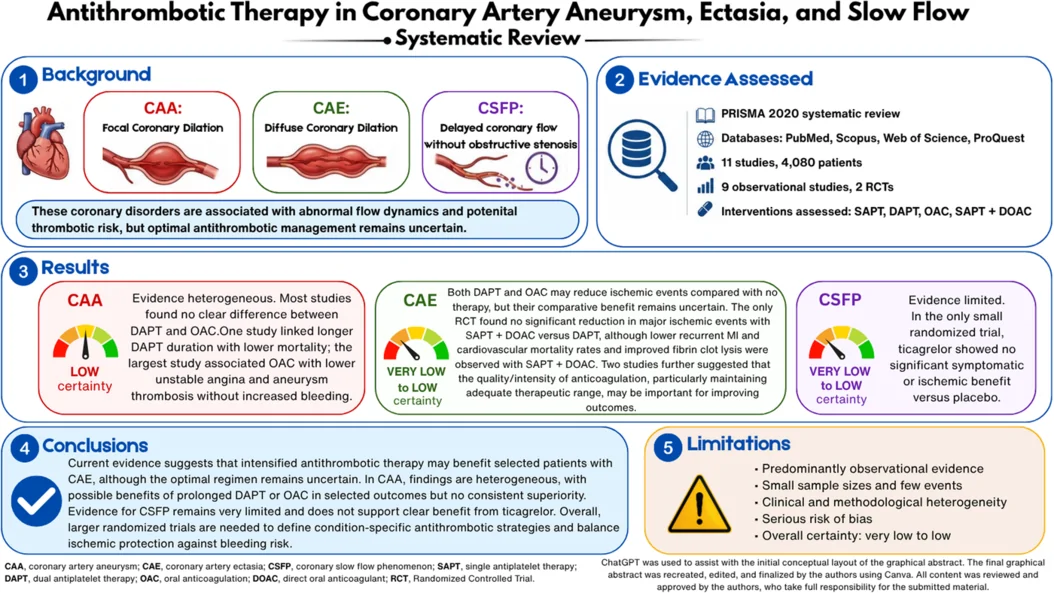

## 1 Introduction

Coronary artery aneurysm (CAA) and coronary artery ectasia (CAE) are rare anatomical cardiovascular conditions characterized by coronary artery dilation exceeding 1.5 times the diameter of the adjacent normal coronary artery. CAA refers to a focal dilation of a coronary artery segment [1], while CAE describes a diffuse dilatation involving more than 50% of the vessel length [2]. The coronary slow flow phenomenon (CSFP) is characterized by delayed distal vessel opacification in the absence of significant epicardial stenosis, typically identified by angiographic TIMI frame counts [3]. Prevalence estimates vary, with CAE observed in 0.3%–4.9% of coronary angiograms [4], CAA in 0.3%–5.3% [5], and CSFP in 1%−7% of diagnostic angiograms [6, 7, 8].

The underlying pathophysiology of CAA and CAE is most often atherosclerotic, though congenital, inflammatory, and iatrogenic causes are recognized [3]. Histopathological studies reveal markedly thickened fibrotic intima with the presence of lipid deposits and depletion of the medial elastic tissue. This degradation of elastic tissue represents the principal etiological factor, in conjunction with persistent vascular inflammation [4]. CSFP, by contrast, is attributed to microvascular endothelial dysfunction, heightened vasomotor tone, and small vessel structural remodeling [9]. These mechanisms create an environment conducive to blood stasis and turbulence, predisposing to thrombus formation [10].

Clinically, these entities present along a wide spectrum. Some patients remain asymptomatic, while others develop exertional angina, acute coronary syndromes, arrhythmias, or sudden cardiac death [11]. More specifically, CAE is associated with an increased risk of thrombus formation, embolization, coronary spasm, and spontaneous dissection, all of which may contribute to myocardial infarction (MI) [4, 12]. CAA carries a risk of rupture, thrombosis, distal embolization, and progression to MI [13, 14, 15, 16]. In CSFP, recurrent angina and reduced exercise tolerance significantly impair quality of life despite the absence of obstructive lesions [9].

Given the prothrombotic milieu, antithrombotic therapy has been proposed as a cornerstone in the management of these conditions. Single antiplatelet therapy (SAPT) with aspirin or clopidogrel, dual antiplatelet therapy (DAPT), and oral anticoagulants (OAC) such as warfarin or direct OAC (DOAC) are the main strategies studied [10]. Observational studies suggest that intensified antithrombotic strategies (e.g., DAPT or anticoagulation) may be associated with fewer ischemic events than no therapy in CAE, but comparative effectiveness between strategies remains uncertain due to heterogeneity and study design limitations; the only randomized trial in ACS + CAE showed no significant MACE difference for SAPT + DOAC vs DAPT, despite signals on recurrent MI and fibrin clot lysis time. Accordingly, we avoid strong claims of class‐wide superiority [3, 10, 17].

Evidence regarding antithrombotic therapy in CAA also remains inconsistent; while some studies suggest benefit from prolonged DAPT or OAC, others show no significant reduction in adverse cardiovascular outcomes [18, 19, 20, 21]. Data for CSFP remain sparse and largely inconclusive, with the only randomized trial showing no significant improvement in symptoms or ischemia reduction with ticagrelor compared to placebo [9].

Despite increasing recognition of CAA, CAE, and CSFP as distinct yet overlapping coronary pathologies, significant uncertainties remain regarding their optimal antithrombotic management. Current evidence is largely derived from small observational cohorts, heterogeneous in design, endpoints, and patient characteristics, and only a handful of randomized trials exist—none powered to directly compare standard antiplatelet therapy with intensified strategies across these entities. Although CAA and CAE are structural coronary dilative disorders whereas CSFP is a functional coronary flow abnormality, these entities were examined together because all involve disturbed coronary hemodynamics and abnormal flow patterns that may promote thrombosis or ischemia, raising a shared therapeutic question regarding the role of antithrombotic therapy. This framework does not imply pathophysiologic equivalence or a “one‐size‐fits‐all” treatment paradigm; rather, it provides a basis for evaluating whether disorders with distinct mechanisms but overlapping thrombotic risk profiles may have differing or shared implications for thrombosis prevention and ischemic risk reduction.

Therefore, the present systematic review aims to comprehensively synthesize existing evidence on antithrombotic strategies for CAA, CAE, and CSFP, identify key knowledge gaps, and delineate differences in therapeutic efficacy and safety across these conditions in the context of their distinct pathophysiological mechanisms and thrombotic risk profiles.

## 2 Materials and Methods

### 2.1 Search Strategy and Process of Data Collection

This systematic review was performed employing the “Preferred Reporting Items for Systematic Reviews and Meta‐Analyses” (PRISMA) protocol (Figure 2) and was also registered at PROSPERO (International Prospective Register of Systematic Reviews) with ID = CRD420251008532 [22]. A systematic search utilizing the search strategy provided in the Appendix SA was conducted on September 20, 2025. The Web of Science, ProQuest, PubMed, and Scopus databases were systematically searched to identify published randomized controlled trials (RCTs) and observational studies investigating various antithrombotic therapies for CAE and aneurysms. Although Embase and the Cochrane Central Register of Controlled Trials (CENTRAL) were not included, the selected databases were chosen to provide broad coverage of biomedical and multidisciplinary literature. Additionally, the reference lists of included studies were screened to minimize the risk of missing relevant studies. The inclusion criteria were defined as follows: (I) Population: Adult patients over 18 years old with dilated coronary arteries (coronary aneurysms, ectasia) or CSFP (II) Intervention: Anticoagulant therapy (e.g., warfarin, DOACs, heparin) and antiplatelet therapy (e.g., aspirin, clopidogrel); (III) Comparison: Placebo, no therapy, or other therapeutic approaches; and (IV) Outcomes: Thrombotic events, bleeding complications, mortality, symptom improvement, and major adverse cardiac events. Additionally, studies involving patients with non‐coronary conditions (e.g., aortic or peripheral aneurysms), non‐atherosclerotic causes of coronary aneurysms, ectasia, or microvascular dysfunction (e.g., Kawasaki disease, Marfan syndrome) were excluded. Although Kawasaki disease represents a leading cause of CAAs in younger populations, it was excluded due to its fundamentally distinct pathophysiology as an acute vasculitis, as well as differences in disease progression, patient demographics, and standard management strategies compared with atherosclerosis‐related coronary abnormalities [23, 24]. Including such populations would introduce substantial clinical and mechanistic heterogeneity, thereby limiting the internal validity of the review and the ability to draw clinically meaningful conclusions regarding antithrombotic therapy in the primary target population. Animal studies, reviews, editorials, opinion pieces (unless used for reference screening), case reports, and case series were also excluded. Non‐English studies and studies unrelated to antithrombotic therapies were also excluded.

### 2.2 Study Selection and Data Extraction

Following the compilation of records from the aforementioned databases, Rayyan—a web and mobile app for systematic reviews—was used to identify duplicate results and facilitate the screening process [25]. Duplicated records were identified through the automated duplication finder tool provided by Rayyan and subsequently reviewed and approved by one of the authors (A.K.). Next, Two authors (N.K. and H.B.) independently screened titles and abstracts for eligibility. Full‐text articles were then assessed independently by two authors (N.K. and S.G.). Discrepancies at both stages were resolved through discussion with a third author (A.K.). Additionally, the selected studies were collected along with comprehensive metadata, including study titles, countries, timeframes, exposure categories, participant counts, duration of follow‐up, methodological frameworks, demographic details (e.g., sex and age), and history of comorbidities (Table 1).

**Table 1:** Study characteristics.

Author	Population	Study population *N*	Follow‐up duration	Groups	Exposure *N*	Age	Male gender *N* (%)	Hypertension *N* (%)	Diabetes mellitus *N* (%)	Hypercholesterolemia *N* (%)	Smoking *N* (%)
Nunez‐Gil et al. [21]	CAA	256	52 months	Aspirin	243^a^	65.5 ± 12.4	221 (86.3)	166 (64.8)	73 (28.5)	166 (64.8)	171 (66.8)
				OAC	18						
				DAPT	211						
				Triple Therapy (ASA+ clopidogrel+anticoagulation)	6						
Khubber et al. [19]	CAA	458	62 months	DAPT	140	76.9 ± 10.8	105 (75)	N/A	N/A	N/A	N/A
				OAC	75	77.6 ± 10.4	105 (75)				
D'Ascenzo et al. [18]	CAA	1542^b^	median 3 years (1–6)	OAC	195	67 ± 13	145 (74)	150 (77)	55 (26)	109 (56)	110 (57)
				Non‐OAC	390	66 ± 12	287 (74)	307 (79)	96 (25)	227 (58)	220 (56)
Matta et al. [20]	CAA	100	46.2 ± 24 months	SAPT	40	64.9 ± 12.6	82 (82)	62 (62)	25 (25)	52 (52)	28 (28)
				DAPT	30						
				OAC	25						
				None	5						
Doi et al. [26]	CAE + AMI	51	49 months	Warfarin TTR > 60%	8	63 ± 13	43 (85)	38 (75)	15 (29)	24 (47)	44 (86)
				Warfarin TTR < 60% or no Warfarin	43						
Gunasekaran et al. [27]	CAE	317	9.7 ± 2.3 years	DAPT	121	69 ± 12	252 (79)	282 (89)	164 (52)	286 (90)	229 (72)
				OAC	105						
				None	91						
Djohan et al. [28]	CAE + STEMI	36	3 years	DAPT	23	57.1 ± 11.7	33 (91.7)	16 (44.4)	4 (11.1)	18 (50.0)	21 (58.3)
				OAC	3						
				SAPT + OAC	7						
				DAPT + OAC	3						
El‐Naggar et al. [29]	Isolated CAE	79	9 months	DAPT	39	58.59 ± 10.99	29 (74.4)	22 (56.4)	11 (28.2)	N/A	14 (35.9)
				OAC	40	53.80 ± 10.40	30 (75.0)	21 (52.5)	8 (20.0)	N/A	22 (55.0)
Solis‑Jiménez et al. [30]	CAE + ACS	350	1027 days (318–1615)	DAPT	228	59.2 ± 11.9	201 (88.2)	132 (57.9)	58 (25.6)	N/A	130 (57.3)
			1111 days (335–1799)	Anticoagulant (VKA/DOAC) ± Antiplatelet	122	57.6 ± 10.3	107 (88.2)	69 (56.6)	16 (13.1)	N/A	74 (60.7)
Araiza et al. [17]	CAE + ACS	62	12 months	DAPT	32	53.4 ± 10.2	26 (83.8)	13 (40.6)	12 (37.5)	9 (28.1)	12 (37.5)
				SAPT + DOAC	30	58.3 ± 10.6	27 (90)	15 (50)	11 (36.6)	8 (27.5)	12 (40)
Pasupathy et al. [9]	CSFP	20	1 month	SAPT (Ticagrelor)	20	61.5 ± 10.5	12 (60)	10 (50)	2 (15)	11 (55)	11 (55)
				Placebo	20ᶜ						

*Note:* Each study is presented once; multiple rows within a study block represent distinct treatment or exposure groups reported in the original study and should not be interpreted as duplicate study entries.

Abbreviations: ACS, acute coronary syndrome; AMI, acute myocardial infarction; CAA, coronary artery aneurysm; CAE, coronary artery ectasia; DAPT, dual antiplatelet therapy; DOAC, direct oral anticoagulant; N/A, data not available; OAC, oral anticoagulant; SAPT, single antiplatelet therapy; STEMI, ST‐elevation myocardial infarction; VKA, vitamin‐K antagonist.

^a^ Antithrombotic treatment counts in Núñez‐Gil et al. were reported among 248 patients alive at discharge and represent overlapping treatment exposures; therefore, exposure counts should not be summed to derive the total study population;

^b^ For D'Ascenzo et al. the study population reflects patients with available anticoagulation status at discharge (*n* = 1542). Group/Exposure Numbers represent the propensity‐score‐matched weighted analytic sample (non‐OAC *n* = 390; OAC *n* = 195) and should not be summed to derive the total study population;

^c^ Pasupathy et al. used a crossover design; the same 20 analyzed participants received ticagrelor and placebo during separate treatment periods. Therefore, exposure counts overlap and should not be summed to derive the study population.

**Figure 1:**
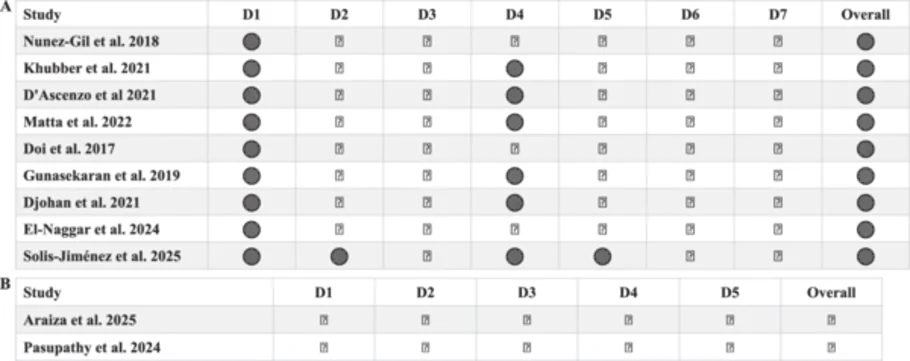
(A) ROBINS‐I risk‐of‐bias assessment of non‐randomized observational studies. D1 = bias due to confounding; D2 = bias in selection of participants into the study; D3 = bias in classification of interventions; D4 = bias due to deviations from intended interventions; D5 = bias due to missing data; D6 = bias in measurement of outcomes; D7 = bias in selection of the reported result; ROBINS‐I = Risk Of Bias In Non‐randomized Studies of Interventions. Overall risk of bias was determined based on the highest level of concern across domains rather than by averaging domain‐level judgments. Green indicates low risk of bias, yellow indicates moderate risk of bias, and red indicates serious risk of bias. (B) Risk‐of‐bias Assessment of Randomized Controlled Trials Using RoB 2.0. D1 = bias arising from the randomization process; D2 = bias due to deviations from intended interventions; D3 = bias due to missing outcome data; D4 = bias in measurement of the outcome; D5 = bias in selection of the reported result. Green indicates low risk of bias, yellow indicates some concerns, and red indicates high risk of bias.

Risk of bias was assessed independently by two authors (M.B. and Z.H.), with disagreements resolved by a third reviewer (A.A.). Observational studies were assessed using the Risk of Bias In Non‐randomized Studies of Interventions tool (ROBINS‐I) [31], while RCTs were assessed using the revised Cochrane Risk of Bias tool for randomized trials (RoB 2) [32] (Table 2). The ROBINS‐I assessment evaluated seven domains: bias due to confounding, selection of participants, classification of interventions, deviations from intended interventions, missing data, measurement of outcomes, and selection of reported results. The RoB 2 assessment evaluated five domains: bias arising from the randomization process, deviations from intended interventions, missing outcome data, measurement of outcomes, and selection of reported results (Figures 1A,B). The certainty of evidence was assessed narratively using a modified GRADE approach [33]. Certainty was evaluated separately for each disease category, including CAA, CAE, and CSFP. The assessment considered risk of bias, inconsistency, indirectness, imprecision, publication bias, and other considerations. The certainty of evidence was summarized as high, moderate, low, or very low (Table 3).

**Table 2:** Risk of bias assessment.

Observational studies	Risk of bias assessment											
	ROBINS‐I											
	D1	D2		D3		D4	D5	D6		D7		Overall
Nunez‐Gil et al. [21]	Serious	Moderate		Moderate		Moderate	Moderate	Moderate		Moderate		Serious
Khubber et al. [19]	Serious	Moderate		Moderate		Serious	Moderate	Moderate		Moderate		Serious
D'Ascenzo et al. [18]	Serious	Moderate		Low		Serious	Moderate	Moderate		Moderate		Serious
Matta et al. [20]	Serious	Moderate		Moderate		Serious	Moderate	Moderate		Moderate		Serious
Doi et al. [26]	Serious	Moderate		Moderate		Moderate	Moderate	Moderate		Moderate		Serious
Gunasekaran et al. [27]	Serious	Moderate		Moderate		Serious	Moderate	Moderate		Moderate		Serious
Djohan et al. [28]	Serious	Low		Moderate		Serious	Low	Moderate		Moderate		Serious
El‐Naggar et al. [29]	Serious	Moderate		Low		Moderate	Moderate	Moderate		Moderate		Serious
Solis‑Jiménez et al. [30]	Serious	Serious		Moderate		Serious	Serious	Moderate		Moderate		Serious

Randomized Controlled Trials	RoB 2.0											
	D1		D2		D3		D4		D5		Overall	
Araiza et al. [17]	Low		Some concerns		Low		Low		Some concerns		Some concerns	
Pasupathy et al. [9]	Low		Low		Some concerns		Low		Some concerns		Some concerns	

*Note:* For ROBINS‐I, D1 indicates bias due to confounding; D2, bias in selection of participants into the study; D3, bias in classification of interventions; D4, bias due to deviations from intended interventions; D5, bias due to missing data; D6, bias in measurement of outcomes; and D7, bias in selection of the reported result. For RoB 2.0, D1 indicates bias arising from the randomization process; D2, bias due to deviations from intended interventions; D3, bias due to missing outcome data; D4, bias in measurement of the outcome; and D5, bias in selection of the reported result. Overall judgments were assigned according to the guidance for each tool.

Abbreviations: RoB 2.0, revised Cochrane Risk of Bias tool for randomized trials; ROBINS‐I, Risk of Bias in non‐randomized Studies of Interventions.

**Table 3:** GRADE certainty assessment of evidence for antithrombotic therapy by disease category.

Certainty assessment									
Category	Study design	No. of studies	Risk of bias	Inconsistency	Indirectness	Imprecision	Publication bias	Other considerations	Certainty of the evidence (GRADE)
CAA	Observational studies	4	Serious concern	Serious concern	Some concern	Serious concern	Undetected but possible	Small samples and heterogeneous study characteristics.	Very low
CAE	5 observational studies and 1 RCT	6	Serious concern	Serious concern	Serious concern	Serious concern	Undetected but possible	Favorable treatment signals were observed, but findings were inconsistent and predominantly derived from non‐randomized studies.	very low to low
CSFP	RCT	1	Some concern	N/A	Serious concern	Serious concern	Undetected but possible	Single RCT with limited generalizability.	Very low to low

Abbreviations: N/A, not applicable; RCT, randomized controlled trial.

### 2.3 Outcomes

#### 2.3.1 Primary Efficacy Outcome

The primary outcome for efficacy was major adverse cardiovascular events (MACE), defined a priori as the time‐to‐first occurrence of cardiovascular death, non‐fatal MI, or non‐fatal ischemic stroke. When studies reported broader composites including unplanned/urgent revascularization, those were recorded as MACCE (MACE plus any cerebrovascular event and/or any repeat revascularization).

#### 2.3.2 Primary Safety Outcome

The primary safety outcome was major bleeding, preferentially abstracted as BARC type 3–5 or ISTH major bleeding; when studies used non‐standard definitions, we extracted the study‐defined category and, when possible, cross‐walked it to BARC 3–5/ISTH major for consistency [34, 35].

Key secondary outcomes were all‐cause mortality, cardiovascular mortality, individual MACE components (MI, ischemic stroke), intracranial hemorrhage (ICH), repeated revascularization (PCI/CABG; target‐vessel/lesion when available), clinically relevant minor, major and total bleeding [36], and also condition‐specific outcomes:

- CAA: aneurysm thrombosis (in‐segment), distal embolization, aneurysm size/progression.
- CAE: ACS recurrence, thrombus burden when reported, and outcomes stratified by Markis class (I–IV).
- CSFP: change in cTFC, angina burden (e.g., SAQ), exercise capacity, and ischemia on testing.

### 2.4 Data Synthesis

**Figure 2:**
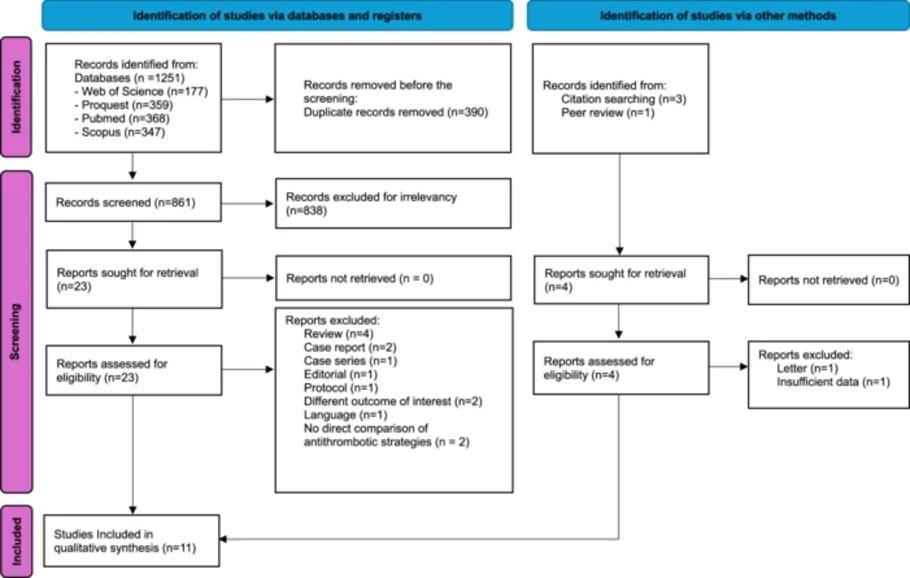
PRISMA 2020 flow diagram of the study selection process [22].

Because fewer than three clinically comparable studies informed each key comparison within each condition (CAA, CAE, or CSFP), we did not perform statistical pooling. Instead, we conducted a structured narrative synthesis following the Systematic Review without Meta‐analysis (SWiM) reporting guideline and PRISMA‐2020 (Figure 2). We (i) grouped studies by condition and antithrombotic strategy (SAPT, DAPT, OAC, SAPT + DOAC); (ii) specified standardized outcome metrics (Hazard ratio for MACE/MACCE and BARC 3–5/ISTH major bleeding; continuous outcomes as mean differences); (iii) applied a common decision rule for effect direction (benefit, harm, or no clear difference) based on adjusted estimates where available and 95% CIs; (iv) presented findings in direction‐of‐effect tables and study‐level summaries; (v) investigated heterogeneity qualitatively (population, definitions, follow‐up, clinical context such as ACS vs chronic, Markis class for CAE, aneurysm size/morphology for CAA, and cTFC for CSFP); (vi) prioritized longest follow‐up and adjusted over crude estimates; (vii) conducted sensitivity analyses excluding abstract‐only evidence and high‐risk‐of‐bias studies; and (viii) assessed certainty narratively using a modified GRADE approach; and (ix) limitations of synthesis methods and how they affect the results and conclusion (Tables S1 & S2).

## 3 Results

The database search identified 1251 records. After removal of 390 duplicates, 861 records were screened, of which 838 were excluded. Twenty‐three reports identified through database searching were assessed for eligibility. In addition, four reports were identified through other methods, including three through citation searching and one during peer review. Following full‐text assessment and application of the eligibility criteria, 11 studies [9, 17, 18, 19, 20, 21, 26, 27, 28, 29, 30] were included in the qualitative synthesis. Of the included studies, nine were non‐randomized observational studies, and two were RCTs.

Risk‐of‐bias assessment indicated an overall serious risk of bias among the nine non‐randomized studies. The two RCTs were judged as having some concerns overall. The full assessments are shown in Figures 1A,B. The modified GRADE assessment showed very low certainty of evidence for CAA and very low to low certainty for CAE and CSFP. This limited certainty was mainly attributable to the predominance of observational study designs, serious risk of bias, clinical and methodological heterogeneity, imprecision due to small sample sizes or few events, and the absence of statistical pooling. The certainty‐of‐evidence assessment is summarized in Table 3.

Of the included studies, six studies comprising 895 participants evaluated antithrombotic therapy in patients with CAE [17, 26, 27, 28, 29, 30]. Four studies, comprising 2356 participants across the included CAA study populations investigated the efficacy of antithrombotic treatment in coronary aneurysm patients [18, 19, 20, 21], and data from one study on 20 patients demonstrated the antithrombotic therapy in CSFP [9]. The main study outcomes, including disease category, antithrombotic strategy, primary findings, and direction of effect, are summarized in Table 4.

**Table 4:** Study outcomes.

Author	Population	Country	Study design	Outcome
Nunez‐Gil et al. [21]	CAA	Spain	Prospective observational	Longer DAPT duration was independently associated with lower mortality (*p* = 0.038). Aspirin use at discharge was independently associated with a lower risk of study‐defined MACE (*p* = 0.047). OAC was not significantly associated with mortality or MACE.
Khubber et al. [19]	CAA	USA	Retrospective cohort	Neither DAPT nor OAC provided significant benefit in reducing MACCE rates.
D'Ascenzo et al. [18]	CAA	Multiple Countries^a^	Observational	OAC significantly reduced unstable angina (*p* = 0.005) and aneurysm thrombosis (*p* = 0.030), while DAPT did not; the benefit of OAC was most pronounced in patients not receiving DAPT.
Matta et al. [20]	CAA	France and Lebanon	Retrospective cohort	No clear advantage of OAC over SAPT or DAPT was observed with respect to MACCE rates.
Doi et al. [26]	CAE + AMI	Japan	Observational cohort	Patients with TTR ≥ 60% had no occurrence of MACE (*p* = 0.03 vs. patients with TTR < 60% or without anticoagulation).
Gunasekaran et al. [27]	CAE	United States	Retrospective observational cohort	− The incidence of ACS was significantly lower among patients on OAC compared with those not on OAC (29% vs. 42%, *p* = 0.02). − Patients treated with DAPT had a lower rate of ACS compared with those not receiving DAPT (17% vs. 34%, *p* = 0.03).
Djohan et al. [28]	CAE + STEMI	Singapore	Retrospective cohort	No significant difference was observed in predicting MACE across the different medications.
El‐Naggar et al. [29]	Isolated CAE	Egypt	Prospective cohort study	− Both treatment groups had comparable cumulative events (acute coronary events, target vessel intervention, or cardiac death) during the 9‐month follow‐up. − Kaplan–Meier analysis showed significantly higher event‐free survival in the OAC group beyond the initial 3 months of follow‐up (*p* = 0.01). − Recurrent events (≥ 2) occurred significantly more often in the DAPT group. − Neither group experienced major bleeding events. − Multivariable Cox regression analysis identified DAPT use (vs. OAC, *p* = 0.03) and lower TTR in OAC users (*p* = 0.03) as independent predictors of adverse clinical events beyond 3 months.
Solis‑Jiménez et al. [30]	CAE + ACS	Mexico	Retrospective cohort	− No statistically significant difference was detected in the risk of MACE during the follow‐up period for the patients receiving anticoagulants ± antiplatelets compared to those on DAPT. − The individual components of MACE, which include all‐cause mortality events, reinfarction events, and stroke events, did not show any differences between the two cohorts. − bleeding outcomes were consistent across both groups without any significant differences. However, GUSTO mild bleeding events were notably more frequent in the anticoagulants ± antiplatelets group when compared to the DAPT group (13.1% vs 3.9%). Logistic regression analysis revealed a hazard ratio of 3.19 (95% CI, 1.41—7.23; *p* = 0.005). Nonetheless, moderate and severe GUSTO bleeding events did not present any differences. − NACE did not exhibit any disparities between the groups.
Araiza et al. [17]	CAE + ACS	Mexico	RCT	Patients in the SAPT + DOAC group had lower recurrent MI rates and reduced fibrin clot lysis time (–14.7%, *p* = 0.25).
Pasupathy et al. [9]	CSFP	United Kingdom	RCT	− Ticagrelor did not significantly improve angina symptoms compared with placebo. − Ticagrelor also had no effect on other patient‐reported outcomes, including angina severity, duration, frequency of prolonged episodes, nitrate use, or SAQ/SF‐36 scores. No serious drug‐related adverse events were reported.

Abbreviations: AMI, acute myocardial infarction; CAA, coronary artery aneurysm; CAE, coronary artery ectasia; CSFP, coronary slow flow phenomenon; DAPT, dual antiplatelet therapy; DOAC, direct oral anticoagulant; MACCE, major adverse cardiovascular and cerebrovascular events; MACE, major adverse cardiac events; N/A, data not available; OAC, oral anticoagulant; RCT, randomized controlled trial; SAPT, single antiplatelet therapy; STEMI, ST‐elevation myocardial infarction; TTR%, percent time in therapeutic range.

a Canada, Cuba, Czech Republic, Germany, Italy, The Netherlands, Spain, United States, Uruguay.

### 3.1 CAA

**Figure 3:**
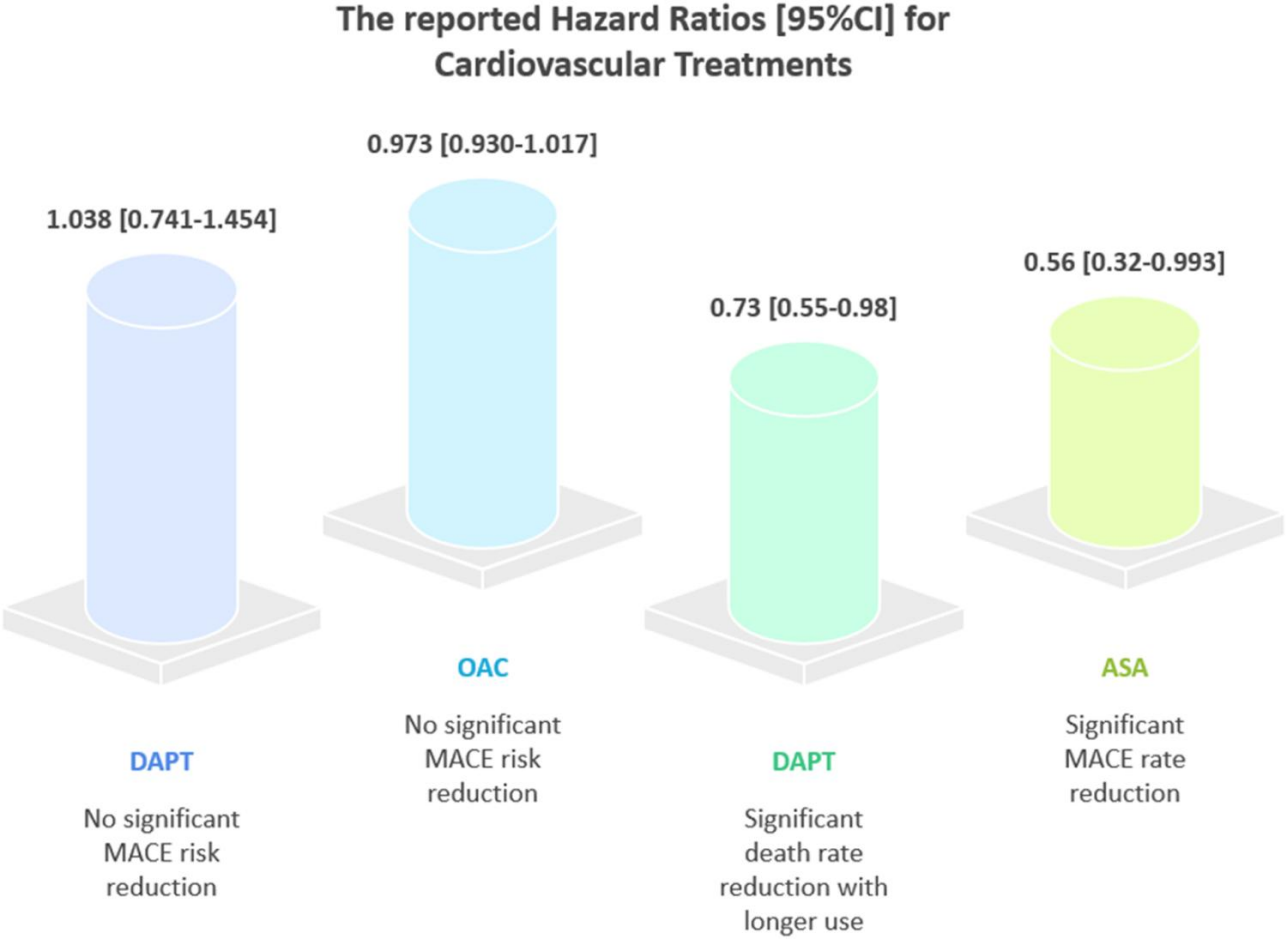
Summary of selected reported hazard ratios for antithrombotic therapy in CAA studies*. *The figure presents HR‐based findings available from Khubber et al. and Núñez‐Gil et al. D'Ascenzo et al. and Matta et al. were incorporated into the narrative synthesis but were not directly plotted because comparable HR estimates for the same antithrombotic outcomes were not consistently reported.

A total of four non‐randomized studies were included in this era. Three of the four studies demonstrated no significant difference between DAPT and OAC, with respect to cardiovascular outcomes, including MACCE and cardiovascular mortality [18, 19, 20]. However, a report in 2018 demonstrated that an increase in DAPT treatment duration can lead to a statistically meaningful reduction of death rates (HR [95% CI]: 0.73 [0.55–0.98], *p* value = 0.038) [21]. In the same study, aspirin use at discharge was independently associated with a lower risk of study‐defined MACE in multivariable analysis (HR 0.56, 95% CI 0.32–0.993; *p* = 0.047). Another study by Fabrizio D'Ascenzo, primarily aimed to evaluate the role of antiplatelet and oral anticoagulation (OAC) therapy in CAA and represented the largest study in these four. D'Ascenzo et al. showed that OAC was associated with significantly lower rates of unstable angina and aneurysm thrombosis, while MI showed a non‐significant downward trend. These findings were not accompanied by a significant increase in bleeding [18]. As the largest included CAA study and one using propensity score matching, it provides important observational support for the potential benefit of OAC in selected patients. Figure 3 summarizes the main hazard ratio findings and overall direction of effect across selected CAA studies.

Taken together, the direction of evidence in CAA is mixed: no consistent superiority of OAC over antiplatelet therapy was demonstrated for major cardiovascular outcomes, although individual studies suggested possible benefits of OAC for unstable angina and aneurysm thrombosis, longer DAPT duration for mortality, and aspirin use for study‐defined MACE. Key sources of heterogeneity included differences in treatment comparisons, outcome definitions, follow‐up duration, analytic approaches, and baseline patient characteristics. These limitations, together with the non‐randomized study designs, result in very low certainty and preclude a definitive treatment preference.

### 3.2 CAE

**Figure 4:**
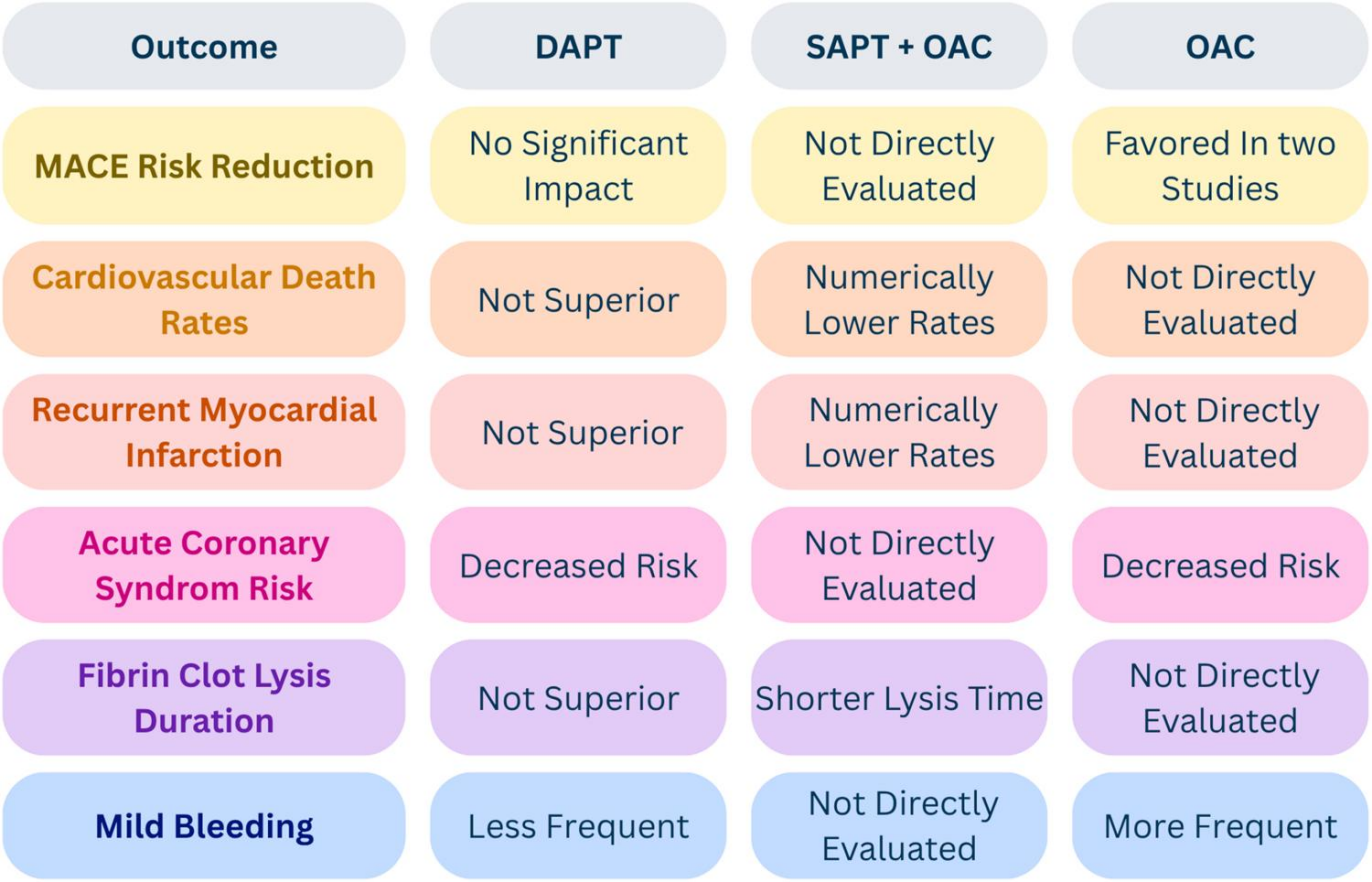
Antithrombotic management results of included studies in CAE.

A total of six studies, including four retrospective studies, one prospective comparative study, and one RCT, were included in this analysis. Among these, five studies compared DAPT with OAC, with or without concomitant antiplatelet therapy, while one study compared either DAPT or OAC versus no antithrombotic therapy. The main outcome‐level findings across the evaluated antithrombotic strategies are summarized in Figure 4. Three of the five comparative studies demonstrated no beneficial effect of OAC on MACE and cardiovascular mortality during follow‐up [17, 28, 30]. The only RCT, conducted by Araiza‐Garaygordobil, did not demonstrate a significant reduction in major ischemic events [17]. Nevertheless, lower rates of recurrent MI (3.3% vs. 12.5%) and cardiovascular mortality were observed in the SAPT plus DOAC group compared with the DAPT group, although these findings were based on a limited number of events and may achieve statistical significance in studies with larger sample sizes. Additionally, SAPT plus DOAC therapy was associated with shorter fibrin clot lysis time.

The other two comparative studies underscored the importance of anticoagulation quality in patients with CAE. El‐Naggar et al. discovered that although the overall adverse events were comparable between the vitamin‐K antagonist (VKA) and DAPT groups throughout the 9‐month follow‐up, the DAPT group experienced a higher rate of adverse events following the initial 3 months. Furthermore, a lower percentage of time in the therapeutic range (%TTR) for VKA patients emerged as an independent predictor of adverse clinical outcomes [29]. Similarly, Doi et al. noted that patients who achieved an optimal %TTR of ≥ 60% did not face MACE, contrasting with those who had %TTR < 60% or who did not undergo anticoagulation therapy [26]. These results imply that, beyond the selection of the antithrombotic agent, the intensity of treatment and the ability to maintain levels within the therapeutic range are vital factors influencing patient prognosis.

In comparisons of antithrombotic therapy versus control, another observational study involving 317 patients demonstrated that both DAPT and OAC, independently, were associated with a reduced risk of acute coronary syndrome over a mean follow‐up period of 9.7 ± 2.3 years. Specifically, acute coronary syndrome occurred in 17% of patients receiving DAPT compared with 34% in the control group (*p* = 0.03), while rates were 29% versus 42% for OAC and control groups, respectively (*p* = 0.02) [27].

Taken together, the overall direction of evidence in CAE suggests a benefit of antithrombotic therapy over no therapy, whereas direct comparisons have not established a consistent advantage of OAC‐based therapy over DAPT. Heterogeneity was driven by differences in clinical setting (isolated CAE, AMI/STEMI, or ACS), regimen composition, anticoagulation quality as reflected by %TTR, follow‐up duration, and outcome definitions, as well as the predominance of non‐randomized studies. Therefore, the apparent benefit of intensified therapy should be interpreted cautiously, and the optimal regimen remains uncertain.

### 3.3 CSFP

Only one study reported an antithrombotic approach in patients with sluggish coronary flow [9]. In this cross‐over clinical trial, which was conducted on 20 patients in 2024, the authors found that ticagrelor, as an antiplatelet agent, did not provide a significant benefit over placebo in reducing angina symptoms over a 4‐week period. Specifically, no significant differences were observed in the frequency, duration, or severity of angina episodes, nor in nitrate consumption or quality‐of‐life indices. No cases of MACE were observed in either group during the study period [9].

Overall, the available evidence does not support a clear benefit of ticagrelor for CSFP; however, the evidence base is limited to a single small crossover RCT. The main sources of uncertainty are the very small sample size, short 4‐week treatment periods, and emphasis on angina and patient‐reported outcomes rather than thrombotic events or long‐term cardiovascular outcomes. Because only one study was available and no MACE events occurred, between‐study heterogeneity could not be assessed and the overall direction of evidence remains inconclusive.

## 4 Discussion

### 4.1 CAA

CAA remains a rare but clinically significant entity, often detected incidentally during coronary angiography [18, 20]. It is associated with complications such as rupture, increased thrombotic risk, distal embolization, and myocardial ischemia [1, 20]. Considering these clinical risks, the optimal antithrombotic strategy in CAA remains uncertain.

The present review found that while OAC and prolonged DAPT are frequently used in CAA, the evidence for their superiority over SAPT is inconsistent. For example, D'Ascenzo et al. demonstrated a reduction in thrombosis and recurrent ischemia with OAC in CAA patients over a 3‐year follow‐up, particularly among those not receiving concomitant DAPT [18]. In contrast, Núñez‐Gil et al. [21] found that longer DAPT duration was associated with improved survival and that aspirin use at discharge was associated with a lower risk of study‐defined MACE, whereas OAC failed to provide significant protection against MACE or all‐cause mortality in aneurysmal patients [21]. Similarly, Khubber et al. reported that neither DAPT nor OAC significantly reduced MACCE rates in CAA, underscoring the variability of outcomes across observational cohorts [19]. Conversely, Matta et al. [20] reported no significant differences in MACCE‐free survival among the evaluated treatment strategies, including medical, percutaneous, and surgical approaches. Additionally, no benefit of OAC over antiplatelet therapy was observed [20].

These conflicting findings likely reflect both clinical heterogeneity among CAA patients and differences in study design. Mechanistically, CAA predisposes to thrombus formation via altered shear stress, endothelial injury, and blood stasis [3]. These hemodynamic conditions resemble those in large vessel aneurysms, such as abdominal aortic aneurysms, where thrombus organization and embolization contribute to morbidity. However, the optimal pharmacologic strategy to mitigate these risks in CAA remains undefined due to the scarcity of RCTs. Most available evidence comes from observational registries, which are inherently limited by selection bias and unmeasured confounders [10]. Consequently, current management is not guided by high‐level evidence but rather relies on extrapolation from pathophysiological principles and individualized risk assessment, highlighting the need for more robust prospective data.

### 4.2 CAE

CAE patients are also susceptible to thrombus formation due to the combination of turbulent flow and endothelial injury [37]. Evidence from observational studies suggests that both DAPT and OAC have been associated with reductions in adverse cardiovascular events compared with no therapy [27], suggesting that more aggressive antithrombotic regimens may be beneficial in appropriately selected patients. Some studies have reported fewer adverse effects with OAC beyond the initial 3 months of therapy [29], whereas others have found no significant difference in MACE rates between DAPT and OAC [28]. Similarly, another observational study comparing DAPT with anticoagulants with or without concomitant antiplatelet therapy reported no significant difference in MACE, secondary ischemic endpoints, or NACE between groups, although mild bleeding events were more frequent in the anticoagulant‐based group, underscoring the tradeoff between ischemic protection and bleeding risk [30]. Moreover, emerging research suggests that a novel combination of antithrombotic agents may also be effective. Araiza‐Garaygordobil et al. [17] conducted the first and, to date, only RCT in this field, directly comparing DAPT with a new regimen of SAPT plus a DOAC. Their findings did not reveal a statistically significant decrease in major ischemic events; although lower rates of recurrent MI and cardiovascular death were observed in the SAPT + OAC group in comparison with DAPT, these findings were based on a limited number of events. Notably, SAPT + OAC was associated with a reduced fibrin clot lysis time, indicating a possible mechanistic advantage through improved fibrinolysis.

CAE has received greater attention in recent literature, particularly through meta‐analyses evaluating the efficacy of antithrombotic strategies. Our synthesis aligns with Azarboo et al., who reported in a network meta‐analysis that DAPT ranked highest for MACE prevention in CAE patients, followed by aspirin monotherapy and anticoagulation [11]. This finding supports the hypothesis that CAE's thrombotic risk profile may more closely resemble that of post‐PCI patients, where platelet aggregation is a dominant mechanism. Notably, this interpretation is consistent with comparative studies showing no clear superiority of anticoagulant‐based therapy over DAPT, while some reports have suggested a higher burden of minor bleeding with anticoagulant‐based regimens [30]. Nonetheless, other studies suggest that combining SAPT with OAC could offer greater ischemic protection in selected patients, albeit at the expense of increased bleeding risk [10]. Taken together, these findings suggest potential benefit from intensified antithrombotic strategies in selected patients, but the predominantly observational evidence and conflicting results warrant cautious interpretation.

One significant challenge in CAE management lies in the variation of study designs, diagnostic criteria, and patient demographics across different studies. For instance, while certain authors characterize CAE as a dilation that exceeds one‐third of the coronary artery length with a diameter more than 1.5 times that of the adjacent normal segment, others describe ectasia as a dilation impacting over 50% of the vessel length [2, 4]. This inconsistency in definitions leads to variations in patient selection and hampers comparability between studies. Furthermore, the variability in disease morphology, thrombus burden, and presence of concurrent atherosclerotic conditions is likely to impact therapeutic outcomes, thereby emphasizing the necessity for personalized treatment strategies that appropriately weigh thrombotic and hemorrhagic risks. Overall, the existing literature indicates that both OAC and DAPT have been implemented in CAE and may offer advantages in selected patients; however, there remains the need for standardized definitions and well‐designed RCTs to guide optimal management.

### 4.3 CSFP

CSFP remains the least studied of the three entities. Our review identified only one clinical trial, a randomized, placebo‐controlled pilot trial of ticagrelor, which failed to show significant improvements in angina burden or ischemia reduction over 4 weeks [9]. While the absence of MACE events in this small cohort limits conclusions regarding thrombotic protection, the results suggest that platelet inhibition alone may be insufficient in CSFP, where microvascular dysfunction plays a dominant role. Pathophysiological parallels with microvascular angina and endothelial dysfunction in systemic diseases raise the possibility that adjunctive therapies targeting nitric oxide bioavailability or microvascular remodeling could prove beneficial.

### 4.4 Cross‐Condition Themes and Research Gaps

When comparing therapeutic strategies across conditions, several themes emerge. First, signals of benefit from more intensive antithrombotic therapy appear most pronounced in CAE; however, these apparently favorable findings do not translate into high certainty of evidence. Most positive signals were derived from non‐randomized observational comparisons and were therefore vulnerable to confounding by indication, selection bias, and non‐randomized treatment allocation. In addition, the direction and magnitude of effects were inconsistent across studies; populations and treatment regimens differed substantially, and several findings were based on small samples or few clinical events. Importantly, the only randomized trial in CAE did not demonstrate a statistically significant reduction in major ischemic events and was limited by the small number of events. Accordingly, despite signals suggesting possible benefit from intensified antithrombotic therapy in CAE, the GRADE certainty of evidence remains very low to low, and these findings should be regarded as hypothesis‐generating pending confirmation in adequately powered randomized trials. Second, the choice between DAPT and OAC should be guided by individual thrombotic and bleeding risk assessment, with particular caution in elderly patients and those with concomitant anticoagulation indications. Third, heterogeneity in outcome definitions—such as varying MACE composites—complicates pooled analyses and underscores the need for standardized endpoints in future studies [11].

From a mechanistic standpoint, the link between flow disturbances and thrombosis in CAA and CAE is well established, but whether modifying flow characteristics through revascularization or stent‐graft placement can improve outcomes remains unknown. In CSFP, microvascular tone and endothelial health—rather than luminal diameter—are the primary determinants of flow, making systemic vasodilators and microvascular‐targeted therapies potentially more relevant than conventional antithrombotic regimens [3].

Given the heterogeneity and limited number of directly comparable studies, we followed the SWiM (Synthesis Without Meta‐analysis) guideline to deliver a structured narrative synthesis (study grouping, common metrics, transparent effect direction, and precision) rather than unstructured vote‐counting (Table S1). Our findings also point to critical research gaps. Large‐scale, multicenter RCTs directly comparing DAPT, OAC, and combination strategies in CAA and CAE are urgently needed. For CSFP, mechanistic studies exploring the interplay between microvascular function, inflammation, and thrombosis could pave the way for novel therapies. Additionally, registries incorporating advanced imaging modalities—such as coronary CT angiography and intravascular ultrasound—may help refine risk stratification and guide treatment selection.

### 4.5 Clinical Implications and Future Directions

Given the heterogeneity of CAA, CAE, and CSFP, management is best individualized. In CAA and CAE, identifying aneurysmal or ectatic morphology may justify careful thrombotic–bleeding risk appraisal before considering therapy intensification; in CSFP, approaches targeting endothelial or microvascular dysfunction may be more relevant than additional antiplatelet therapy. As evidence remains limited, shared decision‐making and close follow‐up are advisable until higher‐quality data become available. Well‐powered, multicenter RCTs comparing DAPT, OAC, and combination strategies—with long‐term bleeding and survival outcomes—would be valuable, and any resulting evidence might inform more tailored guidance.

## Author Contributions

N.K. conducted the primary systematic search. N.K. and H.A. performed the primary screening, which was confirmed by A.K., N.K. and H.B. performed the secondary title and abstract screening, with discrepancies resolved through discussion with M.K. Full‐text articles were assessed independently by N.K. and S.G., and discrepancies at the full‐text assessment stage were resolved through discussion with A.K. M.B. and Z.H. conducted the risk‐of‐bias assessment, with disagreements resolved by A.A. N.K. and A.K. prepared the tables and figures. N.K., A.K., M.I., A.S., A.I., and S.J. contributed to manuscript writing. A.A.A. and H.B.D. performed the final revision of the manuscript. All authors have read and agreed to the published version of the manuscript.

## Funding

The authors have nothing to report.

## Conflicts of Interest

The authors declare no conflicts of interest.

## Supporting information


Supporting File 1

Supporting File 2

Supporting File 3

## Data Availability

The data are available upon request.
