# Supplementary material for: Antithrombotic Therapy in Coronary Artery Aneurysm, Ectasia, and Slow Flow: A Systematic Review
Source: Clin Cardiol. 2026 Sep 25;49(9):e70471. doi: 10.1002/clc.70471 (PMC13613425; doi:10.1002/clc.70471)
Supplement: Supplementary file 2 [file CLC-49-e70471-s002.docx]

**Supplementary File 1. Search Strategies**

**Web of Science**

TS= ((coronary aneurysm OR coronary artery aneurysm OR coronary ectasia OR dilated coronary arteries OR coronary slow flow OR slow coronary flow) AND (anticoagulation therapy OR anticoagulants OR warfarin OR heparin OR direct oral anticoagulants OR DOAC OR rivaroxaban OR apixaban OR dabigatran OR antiplatelet therapy OR antiplatelet agents OR dual antiplatelet therapy OR platelet aggregation inhibitors OR vitamin K antagonist OR VKA) AND (cardiovascular))

**Proquest**

mainsubject("coronary aneurysm" OR "coronary artery aneurysm" OR "coronary ectasia" OR "dilated coronary arteries" OR "coronary slow flow" OR "slow coronary flow" OR "coronary slow flow phenomenon" OR "microvascular dysfunction") AND noft("anticoagulation therapy" OR "anticoagulants" OR "warfarin" OR "heparin" OR "direct oral anticoagulants" OR "DOACs" OR "rivaroxaban" OR "apixaban" OR "dabigatran" OR "anti-platelet therapy" OR "antiplatelet agents" OR "dual antiplatelet therapy" OR "DAPT" OR "aspirin" OR "clopidogrel" OR "ticagrelor" OR "prasugrel" OR "platelet aggregation inhibitors")

**Scopus**

TITLE-ABS-KEY ( "coronary aneurysm" OR "coronary artery aneurysm" OR "coronary ectasia" OR "dilated coronary arteries" OR "coronary slow flow" OR "slow coronary flow" ) AND TITLE-ABS-KEY ( "anticoagulation therapy" OR "anticoagulants" OR "warfarin" OR "heparin" OR "direct oral anticoagulants" OR "DOACS" OR "rivaroxaban" OR "apixaban" OR "dabigatran" OR "anti-platelet therapy" OR "antiplatelet agents" OR "dual antiplatelet therapy" OR "platelet aggregation inhibitors" OR "Vitamin K antagonist" OR "VKA" ) AND TITLE-ABS-KEY ( "cardiovascular" )

**Pubmed**

("Coronary Aneurysm"[Mesh] OR coronary ectasia[Title/Abstract] OR "dilated coronary segment"[Title/Abstract] OR "dilated coronary"[Title/Abstract] OR "dilated coronary arteries"[Title/Abstract] OR "coronary slow flow"[Title/Abstract] OR "coronary ectasia aneurysm"[Title/Abstract]) AND (anticoagulant[Mesh] OR "Platelet Aggregation Inhibitors"[Mesh] OR warfarin[Title/Abstract] OR "heparin"[Title/Abstract] OR "direct oral anticoagulants"[Title/Abstract] OR doac[Title/Abstract] OR "dabigatran"[Title/Abstract] OR "apixaban"[Title/Abstract] OR rivaroxaban[Title/Abstract] OR "anti platelet therapy"[Title/Abstract] OR "dual antiplatelet therapy"[Title/Abstract] OR "dapt"[Title/Abstract] OR "aspirin"[Title/Abstract] OR "clopidogrel"[Title/Abstract] OR "ticagrelor"[Title/Abstract] OR "prasugrel"[Title/Abstract])
