# Supplementary material for: Antithrombotic Therapy in Coronary Artery Aneurysm, Ectasia, and Slow Flow: A Systematic Review
Source: Clin Cardiol. 2026 Sep 25;49(9):e70471. doi: 10.1002/clc.70471 (PMC13613425; doi:10.1002/clc.70471)
Supplement: Supplementary file 3 [file CLC-49-e70471-s003.docx]

**Supplementary Table 1.** Summary of outcome groupings for SWiM Analysis

| Population | Outcome | Author | Year | Journal | PMID | Reference |
| --- | --- | --- | --- | --- | --- | --- |
| Coronary Artery Aneurysm | MACE + All-Cause Mortality | Nunez-Gil et al. | 2018 | Cardiovasc Revasc Med | 29276176 | 21 |
|  | MACE | Khubber et al. | 2021 | Open Heart | 33568555 | 19 |
|  | MACCE | Matta et al. | 2022 | Rev Cardiovasc Med | 39076619 | 20 |
|  | Unstable angina + Aneurysm thrombosis | D'Ascenzo et al. | 2021 | Catheterization and Cardiovascular Interventions | 32902099 | 18 |
| Coronary Artery Ectasia | MACE | Doi et al. | 2017 | Arteriosclerosis, Thrombosis, and Vascular Biology | 29051141 | 33 |
|  | MACE | Djohan et al. | 2021 | Cardiovasc Revasc Med | 34887205 | 32 |
|  | ACS | Gunasekaran et al. | 2019 | Catheterization and Cardiovascular Interventions | 30393992 | 35 |
|  | MACE + Major Bleeding | El-Naggar et al. | 2024 | Int J Cardiol | 39089480 | 34 |
|  | MI + Fibrin Clot Lysis Time | Araiza et al. | 2025 | American Heart Journal | 39615618 | 17 |
|  | MACE + Bleeding + NACE | Solis‑Jiménez et al. | 2025 | Cardiovascular Drugs and Therapy | 40991139 | 36 |
| Coronary Slow Flow Phenomenon | Angina symptoms | Pasupathy et al. | 2024 | J Clin Med | 39274447 | 9 |

MACE: Major adverse cardiovascular events; MACCE: Major adverse cardiac and cerebrovascular events; NACE: Net Adverse Clinical Events
